# High Levels of Dual-Class Drug Resistance in HIV-Infected Children Failing First-Line Antiretroviral Therapy in Southern Ethiopia

**DOI:** 10.3390/v10020060

**Published:** 2018-02-01

**Authors:** Birkneh Tilahun Tadesse, Natalie N. Kinloch, Bemuluyigza Baraki, Hope R. Lapointe, Kyle D. Cobarrubias, Mark A. Brockman, Chanson J. Brumme, Byron A. Foster, Degu Jerene, Eyasu Makonnen, Eleni Aklillu, Zabrina L. Brumme

**Affiliations:** 1 Department of Pediatrics, Hawassa University, Hawassa 1506, Ethiopia; 2 Faculty of Health Sciences, Simon Fraser University, Burnaby, BC V5A 1S6, Canada; nkinloch@sfu.ca (N.N.K.); bbaraki@sfu.ca (B.B.); kcobarru@sfu.ca (K.D.C.); mark_brockman@sfu.ca (M.A.B.); 3 British Columbia Centre for Excellence in HIV/AIDS, Vancouver, BC V6Z 1Y6, Canada; hlapointe@cfenet.ubc.ca (H.R.L.); cbrumme@cfenet.ubc.ca (C.J.B.); 4 Departments of Dermatology and Pediatrics, Oregon Health Sciences University, Portland, OR 97239, USA; bafoster2@hotmail.com; 5 Management Sciences for Health, Addis Ababa 1250, Ethiopia; degujerene@gmail.com; 6 Department of Pharmacology, College of Health Sciences, Addis Ababa University, Addis Ababa 9086, Ethiopia; eyasumakonnen@yahoo.com; 7 Division of Clinical Pharmacology, Department of Laboratory Medicine, Karolinska Institute, Karolinska University Hospital Huddinge C1:68, 141 86 Stockholm, Sweden; eleni.aklillu@ki.se

**Keywords:** HIV, pediatrics, children, Ethiopia, first-line combination antiretroviral therapy (cART), treatment failure, drug resistance, genotyping, dried blood spots

## Abstract

Clinical monitoring of pediatric HIV treatment remains a major challenge in settings where drug resistance genotyping is not routinely available. As a result, our understanding of drug resistance, and its impact on subsequent therapeutic regimens available in these settings, remains limited. We investigate the prevalence and correlates of HIV-1 drug resistance among 94 participants of the Ethiopia Pediatric HIV Cohort failing first-line combination antiretroviral therapy (cART) using dried blood spot-based genotyping. Overall, 81% (73/90) of successfully genotyped participants harbored resistance mutations, including 69% (62/90) who harbored resistance to both Nucleoside Reverse Transcriptase Inhibitors (NRTIs) and Non-nucleoside Reverse Transcriptase Inhibitors (NNRTIs). Strikingly, 42% of resistant participants harbored resistance to all four NRTIs recommended for second-line use in this setting, meaning that there are effectively no remaining cART options for these children. Longer cART duration and prior regimen changes were significantly associated with detection of drug resistance mutations. Replicate genotyping increased the breadth of drug resistance detected in 34% of cases, and thus is recommended for consideration when typing from blood spots. Implementation of timely drug resistance testing and access to newer antiretrovirals and drug classes are urgently needed to guide clinical decision-making and improve outcomes for HIV-infected children on first-line cART in Ethiopia.

## 1. Introduction

Globally, an estimated 1.8 million children 15 years of age or younger are living with HIV-1, of which >1.5 million reside in Sub-Saharan Africa [1,2]. Ethiopia features a substantial pediatric HIV-1 burden: an estimated 65,100 children are currently living with HIV-1, representing 9% of all infections nationally, and nearly 3200 AIDS-related child deaths occur annually [1,3]. Although combination antiretroviral therapies (cART) have significantly reduced mother-to-child transmission and have improved pediatric HIV clinical outcomes in the developing world [4,5,6,7,8,9,10,11,12,13,14,15], pediatric HIV treatment remains a major challenge, particularly in resource-limited settings, due to the complexities of administering cART and the greater potential for rapid clinical progression in untreated (or inadequately treated) children compared to adults [16,17,18,19,20,21,22,23]. Indeed, rates of first-line HIV pediatric treatment failure in Ethiopia are estimated at 13–19% [24,25].

Clinical monitoring of HIV treatment is also a major challenge in resource-limited settings where, due to the lack of availability of both routine viral load and genotypic drug resistance testing [26], WHO clinical and immunological criteria are typically used to define treatment failure [27]. As a result, regimens are often changed without knowledge of HIV-1 drug resistance [27,28] and the contribution of drug resistance to treatment failure in these settings remains incompletely understood. Although studies of HIV-infected adults failing first-line cART in Africa [29,30,31,32], Asia [33,34] and Central America [35], as well as a meta-analysis across multiple global sites [36], suggest resistance mutation burden is substantial in these populations, studies investigating the burden of drug resistance among children failing first-line cART in resource limited-settings are scarcer [37,38,39,40], thus limiting our knowledge in this area.

In Ethiopia, as in many other resource-limited settings, HIV-1 drug resistance testing is not widely available [28]. Indeed, more than a decade after the widespread rollout of cART in Ethiopia in 2005, the burden of HIV-1 drug resistance among pediatric patients remains largely uncharacterized. To our knowledge, only one previous study reported pediatric HIV-1 drug resistance profiles in specimens collected in the early years following cART rollout in the country [41]. To address this gap, we investigate the burden and characteristics of HIV-1 drug resistance among participants of the Ethiopia Pediatric HIV Cohort (EPHIC) who are experiencing virologic failure of their first-line cART regimen, using dried blood spot-based genotyping [42].

## 2. Materials and Methods

### 2.1. Study Participants

The Ethiopia Pediatric HIV Cohort (EPHIC) was established in 2015 with the primary goal of developing context-specific clinical/immunological prediction rules for the diagnosis of first-line cART failure in HIV-infected children in Southern Ethiopia, where access to viral load monitoring is limited [42]. EPHIC comprises 780 HIV-infected children <18 years of age who were on, or eligible to initiate, first-line cART. Participants were recruited at six clinical centers in Southern Ethiopia. Clinical and sociodemographic characteristics were collected at enrolment (baseline), and all participants were assessed semiannually for first-line cART failure using WHO-defined clinical, immunological and virological criteria [27]. The present study comprised the ninety-four participants of the Ethiopia Pediatric HIV Cohort (EPHIC) who were experiencing virologic treatment failure, defined as a plasma viral load greater than 2500 copies/mL while on first-line cART, between enrolment (if the participant had been on cART for >5 months) and February 2017. Viral loads were determined using the RealTime HIV-1 viral load test (Abbott, Des Plaines, IL, USA).

### 2.2. Ethics Statement

Approval to conduct this study was obtained from the Institutional Ethics Review Boards of Hawassa University College of Medicine and Health Sciences, the Southern Nations, Nationalities and Peoples Region (SNNPR) Regional Health Bureau, and Simon Fraser University (P026-19/1421, 31 July 2015). Blood samples were collected after obtaining written informed consent in accordance with the tenets of the Declaration of Helsinki. For participants ≤ 12 years written informed consent was obtained from their parent or guardian, while, for participants > 12 years of age, consent was obtained from both the participant and their parent/guardian. All informed consent documents were provided in the local language.

### 2.3. Specimen Collection, Handling and Storage

In February/March 2017, up to 5 blood spots (approximately 50 μL each) were collected from each participant by fingerprick on dried blood spot cards (Labmate, Cape Town, South Africa) and dried overnight at room temperature. Each card was individually stored in a plastic specimen bag with desiccant pack and shipped to Simon Fraser University in April 2017. Spots were stored at room temperature until September 2017, after which all remaining blood spots were transferred to −80 °C.

### 2.4. Nucleic Acid Extraction and Viral Genotyping

A standard 1/4″ manual hole punch was used to transfer blood spots into sterile tubes for nucleic acid extraction, the punch was cleaned of residual material between participant cards by punching 10 holes into clean filter paper [43]. Each participant’s blood spots were subjected to up to two nucleic extraction attempts, each using two blood spots as starting material, as follows. For all 94 participants, total nucleic acids were extracted from two blood spots using the PureLink Genomic DNA Extraction Kit (Invitrogen, Carlsbad, CA, USA) according to the manufacturer’s instructions. For 88 participants, total nucleic acids were also extracted from two additional blood spots using the NucliSENS easyMAG Automated Total Nucleic Acid Extraction System (BioMerieux, Marcy-l’Étoile, France).

HIV-1 Protease and a portion of Reverse Transcriptase (RT) spanning at minimum codons 1 through 234 were amplified using PCR with or without an initial RT step, using up to 4 oligonucleotide primer sets (1 primary, 3 alternate) optimized for amplification of various HIV-1 group M subtypes (Supplementary Materials Table S1). Amplification with an initial RT step was performed by RT-PCR using the SuperScript III One-Step RT-PCR System with Platinum Taq High Fidelity DNA Polymerase (Invitrogen) followed by nested PCR using the Expand HiFi System (Roche, Basel, Switzerland). Amplification without an initial RT step was performed by nested PCR using the Expand HiFi System (Roche). Amplicons were bulk (directly) sequenced on either a 3730xl or 3130xl automated DNA sequencer (Applied Biosystems, Foster City, CA, USA). Chromatograms were analyzed using Sequencher version 5.0.1 (Gene Codes, Ann Arbor, MI, USA) or the automated basecalling software RECall [44], where nucleotide mixtures were called if the secondary peak exceeded 25% of the dominant peak height (Sequencher) or 17.5% of the dominant peak area (RECall). Sequences were screened for hypermutation, deletions, frame shifts and stop codons using the Quality Control tool hosted by the Los Alamos HIV Sequence Database (LANL) (https://www.hiv.lanl.gov/content/sequence/QC/index.htmL) and the Stanford Drug Resistance Database quality assessment tool. Sequences were aligned using HIVAlign (options: MAFFT, codon-alignment) [45] and visualized using AliView [46]. Phylogenetic inference was performed by maximum likelihood using PhyML under a General Time Reversible model of nucleotide substitution [47] on nucleic acid alignments trimmed to Protease and the first 234 codons of Reverse Transcriptase. Phylogenies were generated from full alignments as well as alignments stripped of all codons associated with HIV surveillance drug resistance mutations [48] to control for any effects on tree topology. Phylogenies were visualized using FigTree (version 1.3.1). Patristic (i.e., phylogenetically inferred pairwise tip-to-tip distances) were extracted from Newick treefiles using PATRISTIC [49]. HIV-1 subtype was determined using HIV LANL’s Recombinant Identification Program (RIP) [50]. All intact sequences isolated from each participant have been deposited in GenBank (accession numbers MG839286-MG839482).

### 2.5. Drug Resistance Genotyping

The Stanford University HIV Drug Resistance Database HIVdb Program (version 8.4, https://hivdb.stanford.edu/hivdb) was used to investigate the presence of drug resistance mutations conferring resistance to Protease Inhibitors (PIs), Nucleoside Reverse Transcriptase Inhibitors (NRTIs), and Non-Nucleoside Reverse Transcriptase Inhibitors (NNRTIs) [51,52]. Resistance to individual drugs was also defined using the Stanford Drug Resistance database where genotypes exhibiting any level of reduced susceptibility to a given drug were considered “resistant” [51,52]. Each sequence was evaluated individually; in addition, for participants for whom >1 sequence was obtained, an inclusive consensus (i.e., a sequence that incorporates all observed polymorphisms at all sites) was generated and interpreted. This was done to maximally capture the breadth of drug resistance within a given individual as well as to assess the degree of underestimation of drug resistance by a single versus replicate testing approach [53,54,55,56,57,58,59,60]. Hypermutated or otherwise defective sequences were excluded from drug resistance interpretation.

### 2.6. Statistical Analyses

Non-parametric statistical analyses were conducted using R (version 3.4) or GraphPad Prism (version 6, San Diego, CA, USA).

## 3. Results

### 3.1. Patient Characteristics

As of February 2017, a total of 94 (of 780; 12%) EPHIC participants were experiencing virologic failure of first-line cART with a plasma viral load greater than 2500 copies/mL and were therefore included in the present study. Overall, the sociodemographic and clinical characteristics of this subset of participants resembled those of the larger EPHIC cohort [42]. At baseline (cohort enrollment), participants were a median 12 years of age (Interquartile Range [IQR] 9–14 years), 60% were male, and participants had median height and weight-for-age *Z*-scores of −1.3 (IQR −2.1–(−0.4)) and −1.5 (IQR −2.1–(−0.6)), respectively, where a WHO-defined Anthropomorphic *Z*-score between −2 and +2 is considered normal [61] (Table 1). At baseline, the median duration of first-line cART was 35 months (IQR 18–70.5 months), the median CD4+ T-lymphocyte (T-cell) count was 500 cells/mm^3^ (IQR 247–781 cells/mm^3^), and the median viral load was 3.9 log_10_ copies/mL (IQR 3.6–4.4 log_10_ copies/mL). At baseline, the majority (85%) of participants were classified as WHO Clinical Stage 1 [62]. The median viral load at time of failure was 4.2 log_10_ copies/mL (IQR 3.8–4.7 log_10_ copies/mL). All first-line regimens consisted of two Nucleos(t)ide Reverse Transcriptase Inhibitors (NRTI), Lamivudine (3TC) with either Zidovudine (AZT) (66%), Stavudine (d4T) (29%), Abacavir (ABC) (3%) or Tenofovir (TDF) (2%), and one Non-nucleoside Reverse Transcriptase Inhibitor (NNRTI), either Nevirapine (NVP) (77%) or Efavirenz (EFV) (22%). A single individual received PI-based first-line cART with Lopinavir/Ritonavir. Subsequently, 48% of participants switched their initial regimen, in the majority of cases (67%) due to changes in Ethiopia’s National HIV Treatment Guidelines which removed d4T from the list of recommended drugs [63]. Most participants (96%) were not exposed to ART prophylaxis for prevention of mother-to-child transmission.

### 3.2. HIV-1 Genotyping from Dried Blood Spots

A total of 197 intact, non-hypermutated HIV-1 Protease and Reverse Transcriptase sequences were obtained from dried blood spots from 90 of 94 participants (median 2 (IQR 2–3, maximum 5) sequences/participant). This translates to a 96% success rate, which is consistent with other studies utilizing similar approaches [54,64,65,66,67,68,69,70,71]. Sequences isolated from the same participant were phylogenetically related (Supplementary Materials Figure S1), and exhibited a median 1.8 (IQR 1.2–3) amino acid differences per 100 codons (where amino acid mixtures were counted as full differences) and a median patristic distance of 0.01 (IQR 0.006–0.02) substitutions/nucleotide site. No evidence of epidemiologic linkage (manifested as close phylogenetic clustering) was observed between participants. All sequences were subtype C, and were located within a single large sub-lineage that shared a most recent common ancestor with the Botswanan subtype C reference sequence MJ4 [72], consistent with the HIV-1 epidemic in Ethiopia being comprised of a sub-clade of subtype C [73,74,75,76,77,78]. Tree topology was not impacted by the presence of drug resistance codons in the phylogeny (not shown).

### 3.3. Prevalence of HIV-1 Drug Resistance among Ethiopian Children Exhibiting Virologic Treatment Failure of First-Line cART

**Figure 1:**
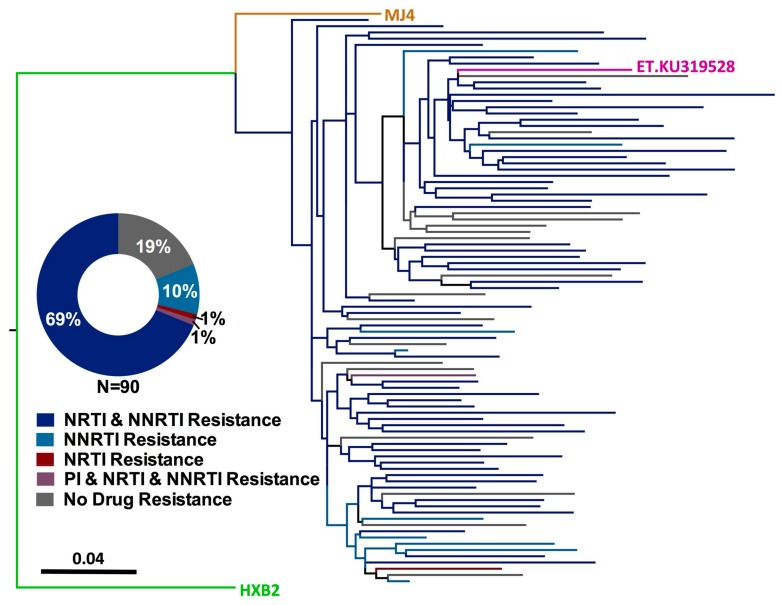
Prevalence of HIV-1 drug resistance among Ethiopian children experiencing virologic failure of first-line cART. Maximum-likelihood phylogeny inferred from the inclusive consensus sequences of 90 participants for whom genotyping was successful. Drug resistance codons were removed from the alignment prior to phylogenetic inference [48]. Scale indicates expected substitutions per nucleotide site. Colours indicate resistance genotype. Reference strains HXB2 (subtype B, green), MJ4 (subtype C-Botswana, orange) and KU319528 (subtype C-Ethiopia, pink) are included. (**Inset**) Distribution of HIV-1 resistance genotypes, stratified by drug class.

The primary objective of our study was to assess the burden of HIV-1 drug resistance among HIV-infected Ethiopian children experiencing virologic failure of first-line cART. As previous studies have shown that drug resistance interpretations based on a single genotype can in some cases underestimate the degree of drug resistance in a given individual [53,54,55,56,57,58,59,60], resistance interpretation was performed on an inclusive consensus sequence constructed from all available intact HIV-1 sequences for each participant (Figure 1). A substantial burden of HIV-1 drug resistance was observed, where 73/90 (81%) participants harbored at least one drug resistance mutation (Figure 1, inset). Resistance to both NRTIs and NNRTIs was most common, with 62 (69%) participants harboring resistance to both drug classes. A single participant harbored resistance to Protease Inhibitors, NRTIs and NNRTIs, 9 (10%) participants harbored resistance to NNRTIs alone, and a single participant harbored NRTI resistance alone (1%). No resistance mutations were observed in 17 (19%) participants, indicating that factors other than drug resistance contribute to virologic treatment failure in this pediatric population.

**Figure 2:**
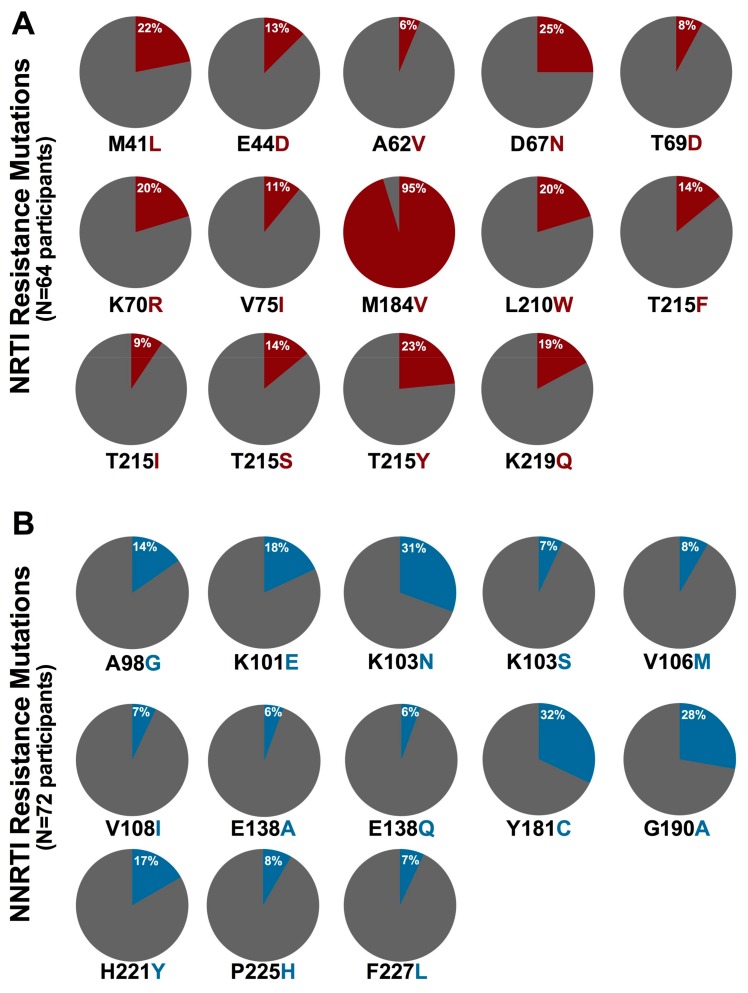
Prevalence of NRTI and NNRTI resistance mutations in participants with resistant genotypes: (**A**) NRTI resistance mutation (red) frequencies among 64 participants with at least one NRTI resistance mutation; and (**B**) NNRTI resistance mutation (blue) frequencies among 72 participants with at least one NNRTI resistance mutation. All mutations observed in >5% of resistant participants are shown.

Participants harboring HIV-1 drug resistance exhibited a broad range of mutations. Currently, 45 NRTI resistance mutations (occurring at 19 RT) codons are defined in the Stanford database [52,79] and/or the IAS-USA list [80]; 28 of these (occurring at 15 codons) were observed in the study participants. M184V was the most commonly observed NRTI resistance mutation, found in 95% (61/64) of participants with NRTI resistance (Figure 2A). This is consistent with the rapid selection of this mutation by 3TC [81,82,83,84] which was included in the first-line regimen of all participants. Next most common were the AZT- and d4T-associated D67N, T215Y, M41L, K70R and L210W mutations [52], which were observed in 25%, 23%, 22%, and 20% of NRTI resistant participants, respectively. Currently, 36 NNRTI resistance mutations (occurring at 18 RT codons) are defined [52,79,80]; 25 of these, occurring at 17 codons, were observed among study participants. Y181C, which confers resistance to all NNRTIs, and K103N, which confers high-level resistance to both EFV and NVP, were observed in 32% (23/72) and 31% (22/72) of NNRTI-resistant participants, respectively (Figure 2B) [52,85]. G190A and K101E, both of which confer resistance to EFV and NVP [52], were observed in 28% (20/72) and 17% (13/72) of NNRTI-resistant individuals, respectively. The single participant with Protease Inhibitor resistance harbored the M46L mutation only; note however this was not the participant who received a PI-based first-line regimen.

Taken together, results reveal diverse mutational profiles among HIV-1-infected children experiencing virologic failure of first-line cART, which generally reflect the specific antiretrovirals, and their relative mutational barriers, used in first-line regimens in the region [52,81,82,83,84,85].

### 3.4. Impact on WHO-Recommended First- and Second-Line Regimens

**Figure 3:**
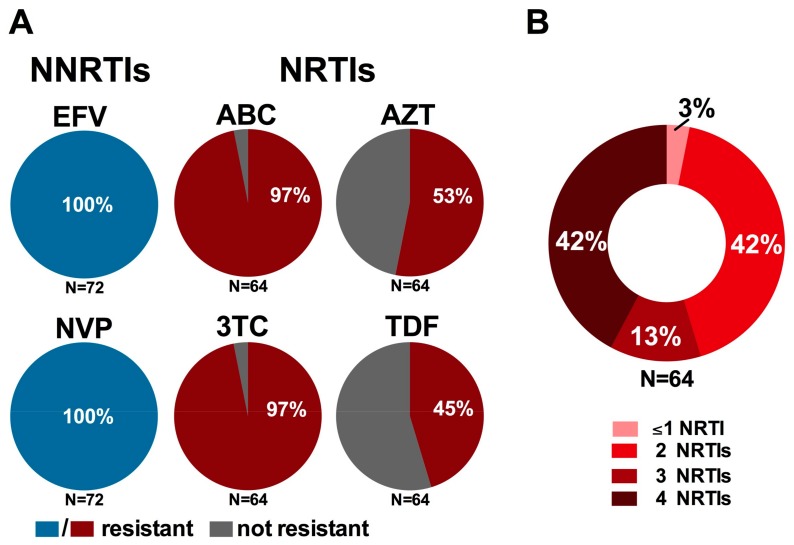
Implications of resistant genotypes on recommended first- and second-line regimens: (**A**) prevalence of resistance to recommended first-line NNRTIs (blue) and NRTIs (red) among participants harboring NNRTI (*N* = 72) and NRTI (*N* = 64) resistance, respectively; and (**B**) burden of multi-NRTI resistance among participants harboring NRTI resistance (*N* = 64). Resistance to individual drugs was defined using the Stanford Drug Resistance Database, where genotypes exhibiting any level of reduced susceptibility to a given drug were considered “resistant” [51,52].

In accordance with WHO guidelines, Ethiopia currently recommends that first-line cART regimens for children three years and older consist of two NRTIs (3TC with either ABC, TDF, or AZT) plus one NNRTI (either EFV or NVP) [27,63], and that second-line regimens consist of two NRTIs (3TC and an additional NRTI not included in the initial regimen) and one Protease Inhibitor (PI) [63]. Consistent with high levels of NNRTI cross-resistance [85], all NNRTI-resistant participants bore resistance to both EFV and NVP (Figure 3A), leaving PIs as the sole second-line option for these children, as recommended in the guidelines.

More concerning however are the implications of NRTI resistance on the success of recommended second-line regimens. Specifically, of the 64 participants harboring NRTI resistance, 62 (97%) were resistant to 3TC (as well as ABC and Emtricitabine (FTC)) by virtue of their carriage of the M184I/V mutation (Figure 3A), while 53% (34/64) and 45% (29/64) of participants with NRTI resistance harbored mutations conferring resistance to AZT and TDF, respectively. While the canonical TDF-resistance mutation K65R was uncommonly observed in this population (3% among NRTI-resistant participants), intermediate to high levels of TDF resistance were instead conferred by combinations of mutations frequently observed together in participants with NRTI resistance, notably K70R, L210W and T215Y/F. While 3TC is recommended for inclusion in second-line regimens based on the known replicative cost of the M184V mutation as well as its ability to increase sensitivity to AZT, d4T and TDF (thus potentially delaying the emergence of mutations conferring resistance to these drugs if regimens are switched in a timely manner), these benefits are gradually eroded in the context of long-term first-line cART failure, especially in cases where mutations to the latter drugs have already been selected [86]. Indeed, 27/64 (42%) NRTI-resistant participants harbored resistance to two of the four NRTIs recommended for use in first- and second-line regimens by both the WHO and Ethiopian National Guidelines, 13% (9/64) carried resistance to three of the four recommended NRTIs and a striking 42% (27/64) harbored resistance to all four drugs (Figure 3B) [27,63]. Taken together, the high burden of resistance mutations observed among participants failing their first-line regimen may substantially compromise the success of recommended second-line regimens, a finding of particular concern as there are currently no recommended third-line therapies for use in Ethiopia [63] and access to additional treatment options is severely limited in sub-Saharan Africa [28,87].

### 3.5. Factors Associated with HIV-1 Drug Resistance among Ethiopian Children Experiencing Virologic Failure of First-Line cART

We next sought to identify sociodemographic and clinical factors associated with drug resistance (classified as any resistance, NRTI resistance and NNRTI resistance) in the study population (Table 2). Participants with NNRTI resistance tended to have been on first-line cART significantly longer than individuals without NNRTI resistance (median 48.5 months [IQR 23–72 months] versus 20.5 [4.8–51.8] months, *p* = 0.03), a trend that also held for participants with NRTI resistance and resistance of any kind. A greater proportion of NRTI-resistant participants had undergone drug substitution compared to those without resistance (57% with drug substitution versus 30%, *p* = 0.04), a trend also observed in participants with NNRTI resistance and resistance of any kind. Consistent with the greater antiviral activity of EFV compared to NVP [88,89], resistance was also less likely to be observed among participants on EFV-based first-line regimens (40% of participants without any resistance were on EFV-based regimens compared to 17% of individuals with resistance of any kind), although this did not reach statistical significance (*p* = 0.08). No significant difference in age, height or weight for age, baseline CD4+ T-cell count, baseline log_10_ pVL, log_10_ pVL at failure, self-reported ART adherence or simultaneous treatment for tuberculosis was observed between individuals with and without HIV-1 drug resistance. Taken together, the identification of longer ART duration and prior regimen changes as correlates of resistance suggest that participants with these traits may warrant closer monitoring. Further, EFV-based NNRTI regimens may reduce the risk of virologic failure with drug resistance in this setting.

### 3.6. Comparison of HIV-1 Drug Resistance Genotypes Obtained with and without an Initial Reverse Transcription Step

In the present study, dried blood spot-based HIV drug resistance genotyping was performed with and without an initial Reverse Transcriptase (RT) step, allowing us to compare the number and distribution of resistance mutations obtained via these two approaches as a secondary objective. A total of 68 (of 94, 72%) study participants had intact HIV genotypes derived from both reaction types. For these, genotypes derived from amplifications with and without the RT step were compared to investigate overall concordance as well as to assess potential bias in detection of resistance mutations in one amplification type over the other (for the 26 cases where more than one genotype was available from a given amplification method, one was selected at random for analysis). 

**Figure 4:**
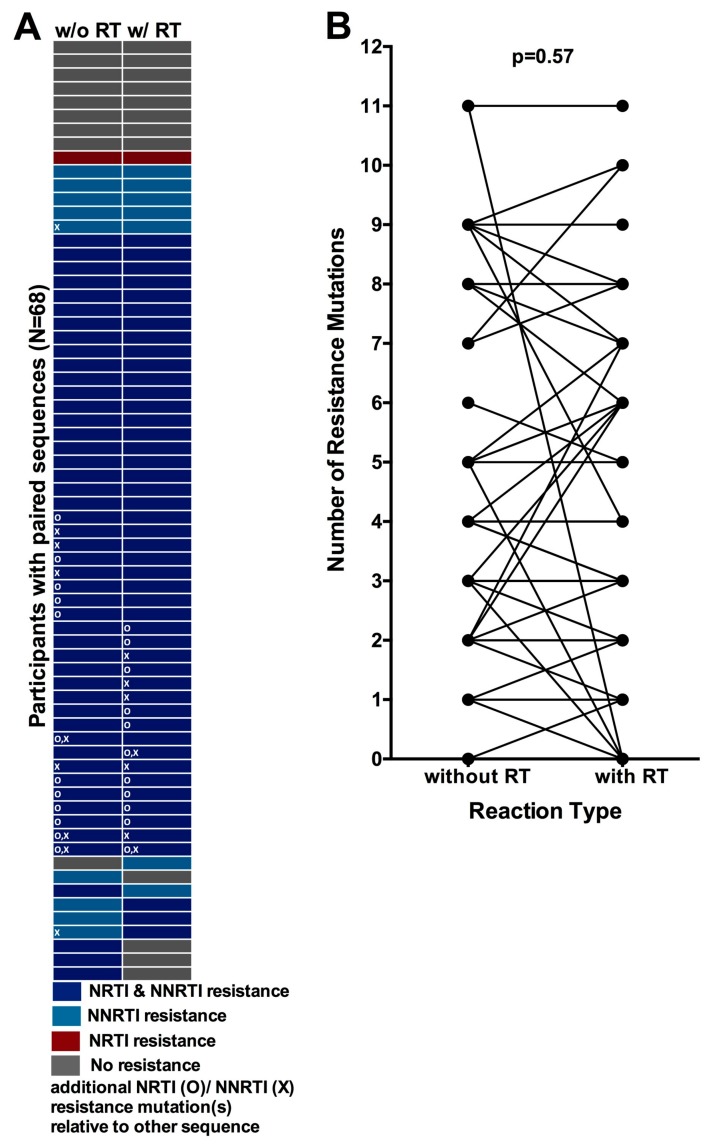
Concordance between resistance genotypes amplified with and without an initial Reverse Transcriptase (RT) step: (**A**) Resistance genotype concordance for 68 participants for whom paired PCR and RT-PCR genotypes were available (one participant per row). Color denotes resistance at the level of drug class. “O” symbols indicate that the genotype harbors at least one NRTI resistance mutation that is not observed in its associated pair, while “X” symbols indicate that the genotype harbors at least one NNRTI resistance mutation that is not observed in its associated pair; (**B**) Total number of resistance mutations observed in genotypes obtained with (right) and without (left) an initial RT step, where connecting lines indicate linked pairs.

Of the 68 paired genotypes, eight consistently exhibited no resistance mutations, while the remaining 60 exhibited at least one resistance-associated mutation in at least one genotype (Figure 4A). Overall, 87% (59/68) of paired genotypes obtained with or without an initial RT step were concordant at the level of drug class. However, only 49% (33/68) of paired genotypes were concordant at the individual mutation level. Furthermore, of the 51% (35/68) of paired genotypes that were discordant at the individual mutation level, these differences affected the resistance genotype at the individual drug level in 18/35 (51%) cases (not shown). Of note, discordances included four cases where drug resistance mutations were detected in genotypes obtained via PCR but not in those amplified using RT-PCR, including one instance where 11 resistance mutations were detected in the PCR-derived sequence while none were observed in the RT-PCR-derived sequence. Paired sequences for these participants are phylogenetically related (Supplementary Materials Figure S1), indicating that discordances are not attributable to sample mix-ups or contamination. Rather, this observation is consistent with the presence of archival proviral DNA harboring resistance mutations even after circulating plasma HIV strains have reverted to susceptible forms following treatment interruption [91,92].

Despite the observation of some discordances however, no significant bias was observed with respect to one amplification method systematically detecting more resistance mutations than the other: the median (IQR) number of mutations detected with and without an RT step was 3 (0–5) and 3 (1–5) respectively (*p* = 0.57, Wilcoxon matched pairs test, Figure 4B). Similarly, no bias was observed when restricting the analysis to only the *N* = 60 paired genotypes from participants exhibiting drug resistance (also *p* = 0.57, not shown). Furthermore, the distribution of individual mutations did not differ between sequences obtained using different amplification methods (all *p* > 0.05, not shown).

Together, these results indicate that, in the studied panel of dried blood spots, detection of drug resistance mutations was not systematically biased by inclusion or exclusion of an initial RT step. Rather, we interpret our results to indicate that HIV drug resistance genotypes derived from a single amplification from blood spots, regardless of method, may underestimate the total breadth of drug resistance present within an individual.

### 3.7. Quantification of the Degree of Underestimation of Resistance from Single versus Replicate Genotypes from Dried Blood Spots

**Figure 5:**
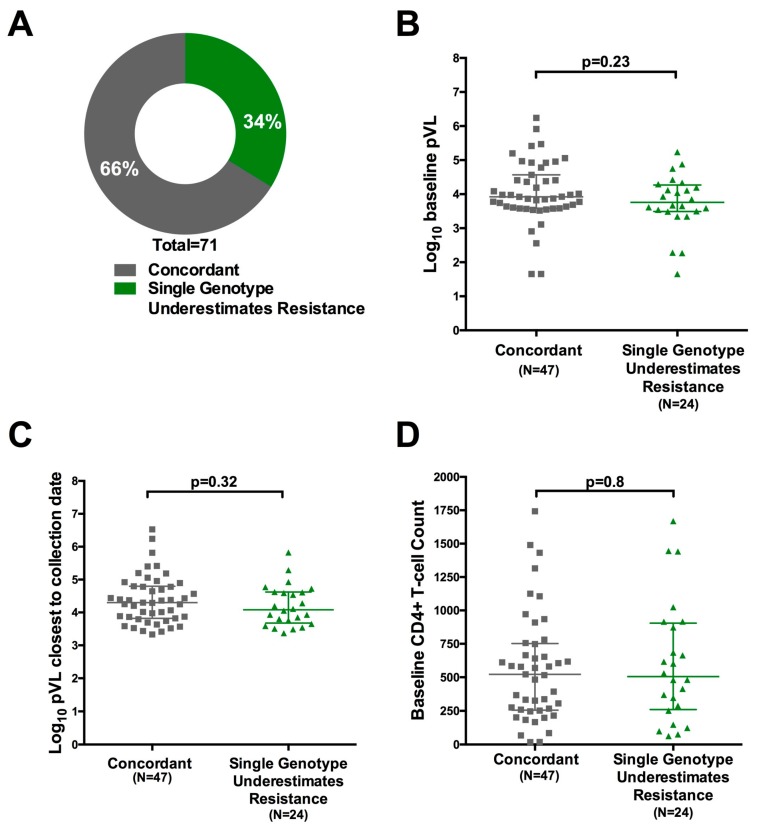
Impact of replicate genotyping on resistance interpretation: (**A**) Proportion of individuals for whom all replicate resistance genotypes were concordant at the individual drug level (grey) versus those where interpretation based on a single genotype would have underestimated the degree of resistance (green). Analysis was based on 71 participants for whom at least two replicate genotypes, derived from any amplification type, were available; (**B**) Log_10_ baseline plasma viral load of participants for whom all replicate resistance genotypes were concordant (grey) versus discordant (green) at the individual drug level; (**C**) Same as (**B**); but for log_10_ plasma viral load at failure (**D**) same as (**B**), but for CD4+ T-cell count at baseline.

We therefore sought to quantify the degree of underestimation of resistance from a single versus replicate genotypes from dried blood spots. For the 71 study patients for whom more than one genotype (amplified by any method) was available, we compared the drug resistance genotypes obtained from each genotype individually to that obtained from their inclusive consensus sequence (Figure 1). For 66% (47/71) of participants, all of their individual genotypes (and thus their inclusive consensus sequence) were concordant at the individual drug level. In 34% (24/71) of cases, however, at least one individual genotype exhibited a discordance at the individual drug level such that the breadth of drug resistance captured by the participant’s inclusive consensus sequence exceeded that of at least one of their individual genotypes (Figure 5A). In other words, genotyping based on a single amplicon may have underestimated resistance to at least one drug in 34% of cases. Similar results were observed when this analysis was restricted to only resistant individuals with two or more sequences amplified using an initial RT step, where resistance would have been underestimated in 8/28 (29%) cases (not shown).

No association between detection of additional antiretroviral drug resistance in combined versus single genotypes and log_10_ baseline viral load, log_10_ viral load at failure, baseline CD4 T-cell count or CD4 T-cell count at time of cART initiation was observed (Figure 5B–D and not shown). Further, no association was observed between underestimation of resistance by a single genotype and total number of genotypes performed (Mann-Whitney *p* = 0.78; not shown), suggesting that this effect is not driven by the collection of >2 sequences for 28 individuals. Overall, results indicate that, when genotyping from blood spots, performing replicate genotypes (i.e., amplifying and sequencing each sample twice) may yield a more complete HIV-1 drug resistance profile regardless of pVL and CD4 count.

## 4. Discussion

The present study, to our knowledge the first to characterize HIV-1 drug resistance in the setting of pediatric first-line treatment failure in Ethiopia in the second decade following cART rollout in this country, reveals a striking 81% prevalence of drug resistance in this population. Moreover, this figure can be interpreted as a lower-bound estimate, as dried blood spot-based genotyping has limited sensitivity to detect the newest emergent resistance mutations relative to that from blood plasma [93]. Our findings starkly contrast the only other report of drug resistance among children failing first-line cART in Ethiopia, undertaken on specimens collected from 13 participants only three years following cART rollout, where only one child harbored HIV-1 drug resistance [41]. Our findings are comparable to rates of overall drug resistance reported in pediatric first-line treatment failure in other African nations including Cote d’Ivoire and Burkina Faso (75%) [38], Mali (73%) [37] and slightly lower than those reported in Cameroon (90%) [39]. Dual-class resistance was most commonly observed in our cohort (69%), an observation consistent with reports from settings where similar pediatric first-line regimens are currently used (e.g., Mali and Cameroon) [37,39,94,95].

Given the complexities of antiretroviral therapy management in the pediatric setting [16,17,18,19,20,21], the prevalence and correlates of drug resistance development are likely to differ between children and adults, but knowledge in this area is limited. Indeed, the prevalence of drug resistance in the present study (81%) is substantially higher than that reported in Ethiopian adults failing first-line regimens (40%) [32]. To our knowledge, ours is the first study to investigate correlates of drug resistance among children failing first-line cART; specifically, we identified duration of cART, prior regimen switching and use of NVP (rather than EFV) as correlates of drug resistance mutation detection. These observations contrast somewhat those from first-line treatment failure among adults in neighboring regions: for example, in a Kenyan study, poor adherence, high viral load, and younger age were associated with first-line cART failure with drug resistance [31]. We acknowledge that a limitation of the present study is that adherence was measured only by self-report, and therefore likely overestimates actual adherence levels. Nevertheless, our findings, taken together with our observation that 19% of children in the study group failed cART without any evidence of drug resistance, further highlight the unique and complex needs of HIV-infected children receiving cART in resource-limited settings [17] and underscores the urgent need to implement timely drug resistance testing to guide clinical decision-making in this population.

Our results also have profound negative implications for the success of second-line pediatric regimens currently recommended in Ethiopia. Currently, the National HIV Treatment Guidelines recommend that the NRTIs included in the second-line regimen consist of 3TC (carried over from the first-line regimen) plus either ABC, AZT, or TDF (whichever was not already included in the initial regimen) [63]. However, 42% of NRTI-resistant participants (and 30% of participants overall) harbored resistance to all four of these NRTIs, meaning that there are effectively no remaining cART options for these children. Expanded and affordable access to newer antiretrovirals and additional drug classes, particularly Integrase Inhibitors, is thus urgently needed in this setting.

Although blood plasma remains the gold standard sample type for HIV-1 drug resistance testing, plasma storage and shipment presents numerous challenges in resource-limited settings [96]. Dried blood spot-based HIV resistance genotyping, which can yield highly concordant results to plasma-based genotyping [64,65,67,68,69,70,71], is thus often used as an alternative. One concern with the use of dried blood spots however is the potentially substantial contribution of proviral DNA to resistance genotypes obtained from this sample type [64,67,69,70]. To mitigate this, the WHO recommends use of RT-PCR for blood spot-based genotyping [96], but direct comparisons between genotypes obtained from dried blood spots with and without an initial RT step are limited [66,67]. As a secondary objective, therefore, we investigated this in a subset of 68 participants for whom paired genotypes, undertaken with and without an RT step, were available. Overall, our results revealed strong (87%) concordance at the level of drug class and good (74%) concordance at the individual drug level, with no systematic bias towards detection of drug resistance mutations (individually, or overall) using one amplification method versus another. This suggests that proviral DNA-based genotyping did not underestimate resistance mutation burden in this cohort. In fact, we observed four instances where drug resistance mutations were detected by nested PCR, but not when an initial RT step was used, suggesting that resistance mutations were present within archived proviral DNA but not circulating viral RNA at the time of sample collection. Rather, our results indicate that replicate genotyping, regardless of amplification method, increased the breadth of resistance detected from blood spots, an observation that corroborates the notion that a single genotype may underestimate the full within-host burden of HIV-1 drug resistance mutations [53,54,55,56,57,58,59,60]. This may be of particular importance for dried blood spots where the input HIV-1 copy number is low. Participants in the present study had a median viral load at failure of approximately 16,000 copies/mL; we therefore estimate that roughly 80 HIV-1 copies were present in each amplification. Further, we were unable to identify any clinical characteristics of individuals for whom replicate genotyping yielded a greater breadth of drug resistance. As such, we suggest that drug resistance testing protocols consider replicate genotyping when using blood spots, if possible.

Effective clinical care of HIV-infected children is paramount in the fight to end HIV/AIDS. Our study reveals a substantial (81%) burden of HIV-1 drug resistance among Ethiopian children failing their first-line regimens, which will greatly limit future treatment options. In fact, for 30% of the children studied, not a single fully active NRTI remains. Access to expanded antiretroviral treatment options and the implementation of routine and timely drug resistance testing in Ethiopia and other resource-limited settings is urgently needed to guide clinical decision-making, to improve prognosis for these vulnerable children, and to meet their basic human rights.

## Figures and Tables

**Table 1:** Clinical and sociodemographic characteristics of EPHIC participants experiencing virologic failure of first-line cART (*N* = 94).

Variable				Summary Statistic	*N*
Age at baseline, years, Median (IQR)				12 (9–14)	94
Gender, % male				60%	94
pVL at baseline, log_10_ copies/mL, Median (IQR)				3.9 (3.6–4.6)	94
pVL at failure, log_10_ copies/mL, Median (IQR)				4.2 (3.8–4.7)	94
CD4+ T-cell count at baseline, cells/mm^3^, Median (IQR)				500 (247–781)	92
CD4+ T-cell count at ART initiation, cells/mm^3^, Median (IQR)				276 (163–613)	89
ART duration at baseline, months, Median (IQR)				35 (18–70.5)	89
Weight for age at baseline, *Z*-score *, Median (IQR)				−1.5 (−2.1–(−0.6))	89
Height for age at baseline, *Z*-score *, Median (IQR)				−1.3 (2.1–(−0.4))	89
combination Antiretrovial T regimen at baseline, % patients					
NRTI					93
		3TC+	AZT	66%	
			d4T	29%	
			TDF	3%	
			ABC	2%	
	NNRTI				93
			EFV	22%	
			NVP	77%	
WHO Clinical Stage at baseline, % patients Stage 1				85%	94

* Height- and Weight-for-age *Z* score was measured using WHO Anthropomorphic Software [61], where a *Z*-score of <−2 is indicative of malnutrition.

**Table 2:** Factors associated with HIV-1 drug resistance in Ethiopian children experiencing virologic failure of first-line cART.

Variable ^1^		NRTI Resistance			NNRTI Resistance			Any Resistance		
		Yes (*N* = 64)	No (*N* = 26)	*p*	Yes (*N* = 72)	No (*N* = 18)	*p*	Yes (*N* = 73)	No (*N* = 17)	*p*
Age at baseline, years, Median (IQR), [*N* = 90]		11 (8.0–14.0)	12.8 (11.0–14.0)	0.07	12.0 (9.0–14.0)	12.0 (9.0–13.0)	0.73	12.0 (9.0–14.0)	12.0 (10.0–13.0)	0.51
Sex, % Male, [*N* = 90]		66%	54%	0.34	60%	72%	0.42	60%	71%	0.58
ART duration at baseline, months Median [IQR], [*N* = 86]		49.5 (23.0–71.8)	25.5 (7.3–61.5)	0.11	**48.5 (23.0–72.0)**	**20.5 (4.8–51.8)**	**0.03**	48.0 (22.5–72.0)	24.0 (6.9–54.5)	0.08
WHO Clinical Stage at baseline, % Stage 1, [*N* = 90]		86%	81%	0.54	86%	79%	0.48	85%	82%	0.72
CD4 count at baseline, cells/mm^3^ Median [IQR], [*N* = 88]		481 (224–752)	483 (270–781)	0.79	481 (248–674)	584 (270–910)	0.50	482 (250–700)	485 (257–957)	0.66
CD4 count at ART initiation cells/mm^3^, Median[IQR], [*N* = 84]		280 (136–646)	265 (195–482)	0.71	245 (149–643)	292 (200–731)	0.44	261 (151–628)	270 (198–581)	0.67
Baseline Weight-for-age *Z*-score * Median[IQR], [*N* = 89]		−1.5 (−2.1–(−0.6))	−1.7 (−2.2–(−0.9))	0.59	−1.5 (−2.1–(−0.7))	−1.8 (−2.4–(−1.2))	0.2	−1.5 (−2.1–(−0.6))	−1.8 (−2.6–(−1.1))	0.31
Baseline Height-for-age *Z*-score * Median[IQR], [*N* = 89]		−1.3 (−2.0–(−0.3))	−1.6 (−2.3–(−0.6))	0.32	−1.3 (−2.1–(−0.4))	−1.5 (−2.1–(−0.6))	0.63	−1.3 (−2.1–(−0.4))	−1.4 (−2.1–(−0.6))	0.77
Baseline ART regimen [*N* = 89]	% AZT-based ^ϕ^	66%	64%	1.00	68%	56%	0.41	67%	59%	0.58
	% D4T-based ^ϕ^	33%	20%	0.31	30%	28%	1.00	29%	29%	1.00
	% NVP-based	81%	71%	0.40	83%	60%	0.08	83%	60%	0.08
	% EFV-based	19%	29%	0.40	17%	40%	0.08	17%	40%	0.08
Drug substitution, %Yes, [*N* = 84]		**57%**	**30%**	**0.04**	55%	27%	0.08	54%	29%	0.14
Adherence to ART, % sub-optimal ^#^, [*N* = 90]		34%	27%	0.62	64%	17%	0.16	36%	18%	0.25
Treatment for Tuberculosis, %Yes [*N* = 90]		33%	31%	1.00	31%	39%	0.58	30%	41%	0.40
Viral load at baseline, Log_10_ copies/mL, Median[IQR], [*N* = 90]		3.8 (3.5–4.4)	3.9 (3.7–4.7)	0.47	3.8 (3.5–4.3)	4.1 (3.8–5.1)	0.16	3.8 (3.6–4.4)	4.0 (3.8–5.1)	0.29
Viral load at failure, Log_10_ copies/mL, Median[IQR], [*N* = 90]		4.2 (3.8–4.7)	4.3 (3.9–4.8)	0.42	4.2 (3.8–4.7)	4.4 (3.9–5.1)	0.20	4.2 (3.8–4.8)	4.8 (3.9–5.0)	0.33

^1^ Associations with *p* < 0.05 are **bolded**. * Height- and Weight-for-age *Z* score was measured using WHO Anthropomorphic Software [61], where a *Z*-score of <−2 is indicative of malnutrition. ^ϕ^ NRTIs used in ≤3 participants were not considered in this analysis. ^#^ Sub-optimal treatment adherence was defined using two self-reported methods: participant recall (number of pills missed in one week and one month) and using the Visual Analogue Scale (VAS) [90].

## Supplementary Materials

The following are available online at http://www.mdpi.com/1999-4915/10/2/60/s1, Figure S1: Maximum-likelihood phylogenetic tree of all intact, non-hypermutated sequences isolated from EPHIC participants experiencing virologic failure of first-line cART; Table S1: Primers used for HIV-1 Protease and Reverse Transcriptase amplification.
