# Supplementary material for: High Levels of Dual-Class Drug Resistance in HIV-Infected Children Failing First-Line Antiretroviral Therapy in Southern Ethiopia
Source: Viruses. 2018 Feb 1;10(2):60. doi: 10.3390/v10020060 (PMC5850367; doi:10.3390/v10020060)
Supplement: Supplementary file 1 [file viruses-10-00060-s001.zip › TadesseEtAl_SupplementalMaterial.pdf]

Supplemental Table 1. Primers used for HIV-1 Protease and Reverse Transcriptase amplification

Primer Set		First Round		Second Round	
		**HXB2 coordinates (start/end)**	**Sequence (5′->3′)**	**HXB2 coordinates (start/end)**	**Sequence (5′->3′)**
**Set 1***	**F**	1979/2005	AAGAAGGGCACMTAGCCARAAAYTGYA	2011/2039	CCTAGGAAAAARGGCTGTTGGAARTGTGG
	**R**	3333/3301	CCACTAACTTCTGTATGTCATTGACAGTCCAGC	3280/3255	ATAGGCTGTACTGTCCATTTATCAGG
**Set 2**	**F**	2008/2031	GCCCCTAGGAAAAAGGGCTGTTGG	2011/2039	CCTAGGAAAAARGGCTGTTGGAARTGTGG
	**R**	3361/3342	TAAATCTGACTTGCCCART	3323/3303	CTGTATRTCATTRACWGTCCA
**Set 3**	**F**	1979/2005	AAGAAGGGCACMTAGCCARAAAYTGYA	2011/2039	CCTAGGAAAAARGGCTGTTGGAARTGTGG
	**R**	3859/3831	GCTCCTACTATGGGTTCTTTYTCYARYTG	3798/3777	CAAACTCCCAYTCAGGRATCCA
**Set 4**	**F**	1992/2015	AGCCAGAAATTGCAGGGCCCCTAG	2074/2095	AGACAGGCTAATTTTTTAGGGA
	**R**	3322/3303	TGTATRTCATTGACAGTCCA	3271/3252	ACTGTCCATTTRTCAGGATG

*** primary primer set**

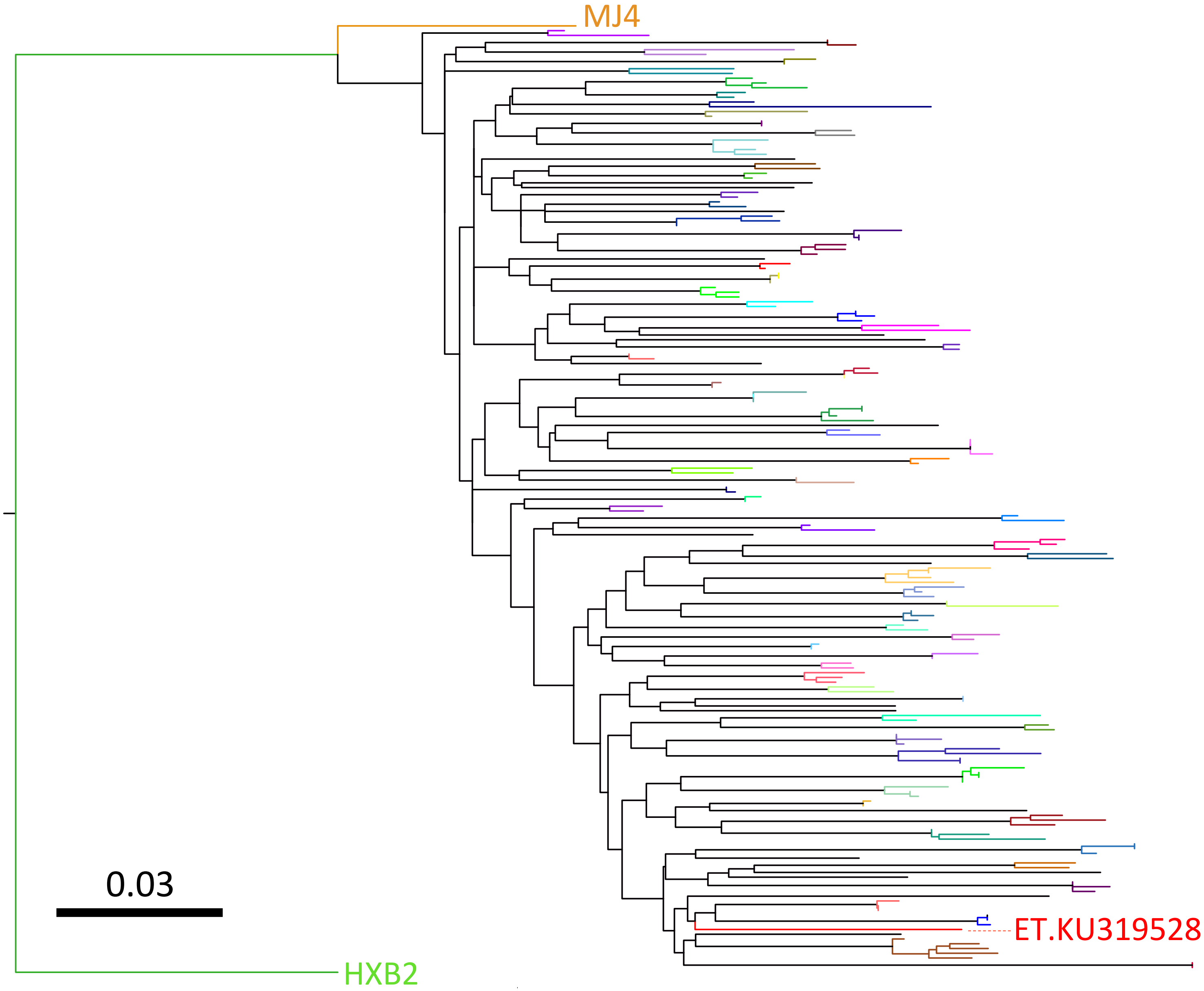

Supplemental Figure 1. Maximum-likelihood phylogenetic tree of all intact, non-hypermutated sequences isolated from EPHIC participants experiencing virologic failure of first-line cART. Scale indicates substitutions per nucleotide site. Drug resistance codons were removed from the alignment prior to phylogenetic inference [48]. Reference strains HXB2 (subtype B, green), MJ4 (subtype C- Botswana, orange) and KU319528 (subtype C- Ethiopia, red) are included. In all cases, replicate sequences from the same participant cluster together.
